# Late Type IIIa Endoleak Despite Adequate Component Overlap after AFX2 EVAR: A Report of Two Cases

**DOI:** 10.3400/avd.cr.26-00144

**Published:** 2026-11-10

**Authors:** Noriyuki Yokoyama, Takamitsu Tatsukawa, Kyokei Fuchizawa, Jeong A Lee, Kouji Kagawa, Daita Kobayashi, Nobuyuki Akasaka, Hideyuki Harada

**Affiliations:** 1 Department of Vascular Surgery, Asahikawa Medical University, Asahikawa, Hokkaido, Japan; 2 Department of Cardiovascular Surgery, Kushiro Kojinkai Memorial Hospital, Kushiro, Hokkaido, Japan; 3 Department of Cardiovascular Surgery, Asahikawa Medical University, Asahikawa, Hokkaido, Japan

**Keywords:** abdominal aortic aneurysm, AFX2 endograft, type III endoleak

## Abstract

Late type IIIa endoleak is a recognized complication of AFX2 (Endologix, Irvine, CA, USA) endovascular aneurysm repair (EVAR). We report 2 cases occurring 6 and 7 years after EVAR despite adequate initial overlap. In Case 1, computed tomography angiography (CTA) did not clearly demonstrate component uncoupling, whereas duplex ultrasonography showed bidirectional (“to-and-fro”) flow and angiography confirmed a type IIIa endoleak. In Case 2, serial computed tomography (CT) retrospectively demonstrated progressive sideways displacement before complete uncoupling. Focal cuff relining and complete endograft relining were performed, respectively. These cases emphasize the need for lifelong surveillance for sac enlargement and changes in endograft configuration after AFX2 EVAR.

## Introduction

Endovascular aneurysm repair (EVAR) has become a standard treatment for infrarenal abdominal aortic aneurysms because of its lower perioperative morbidity and mortality compared with open surgical repair. However, long-term device-related complications remain an important concern, including type III endoleaks caused by separation between modular endograft components. Type III endoleaks expose the aneurysm sac to systemic arterial pressure and therefore require prompt diagnosis and treatment because of the increased risk of aneurysm enlargement and rupture.^1,2)^

The AFX2 endograft (Endologix, Irvine, CA, USA) incorporates an endoskeleton design that provides favorable conformability in patients with a narrow aortic bifurcation. Because proximal sealing frequently requires an extension cuff, the junction between the main body and cuff represents a potential site of late type IIIa endoleak.^3–6)^ Previous reports have described migration and sideways displacement of AFX/AFX2 components, indicating that adequate initial overlap does not necessarily ensure long-term junctional stability.^3–6)^

We report 2 patients who developed late type IIIa endoleaks 6 and 7 years after AFX2 implantation despite adequate initial component overlap. These cases provide longitudinal clinical and imaging observations of a recognized failure mode and highlight the complementary roles of imaging modalities, lifelong surveillance, and individualized secondary intervention.

## Case Report

### Case 1

An octogenarian woman with hypertension, dyslipidemia, and a history of percutaneous coronary intervention had undergone EVAR for a 47-mm infrarenal abdominal aortic aneurysm 6 years earlier. The procedure was performed using an AFX2 main body (25–90/16–30 mm) with a VELA proximal extension cuff (28–90 mm). The overlap between the main body and cuff measured 47 mm. The overlap length was determined on the immediate postoperative contrast-enhanced computed tomography (CT) by counting consecutive 1-mm axial slices showing overlap and was confirmed on sagittal and coronal reconstructed images. Completion angiography and postoperative computed tomography angiography (CTA) demonstrated no evidence of endoleak, with adequate overlap between the main body and proximal cuff (**Fig. 1A**). The patient was discharged uneventfully on postoperative day 10.

**Fig. 1:**
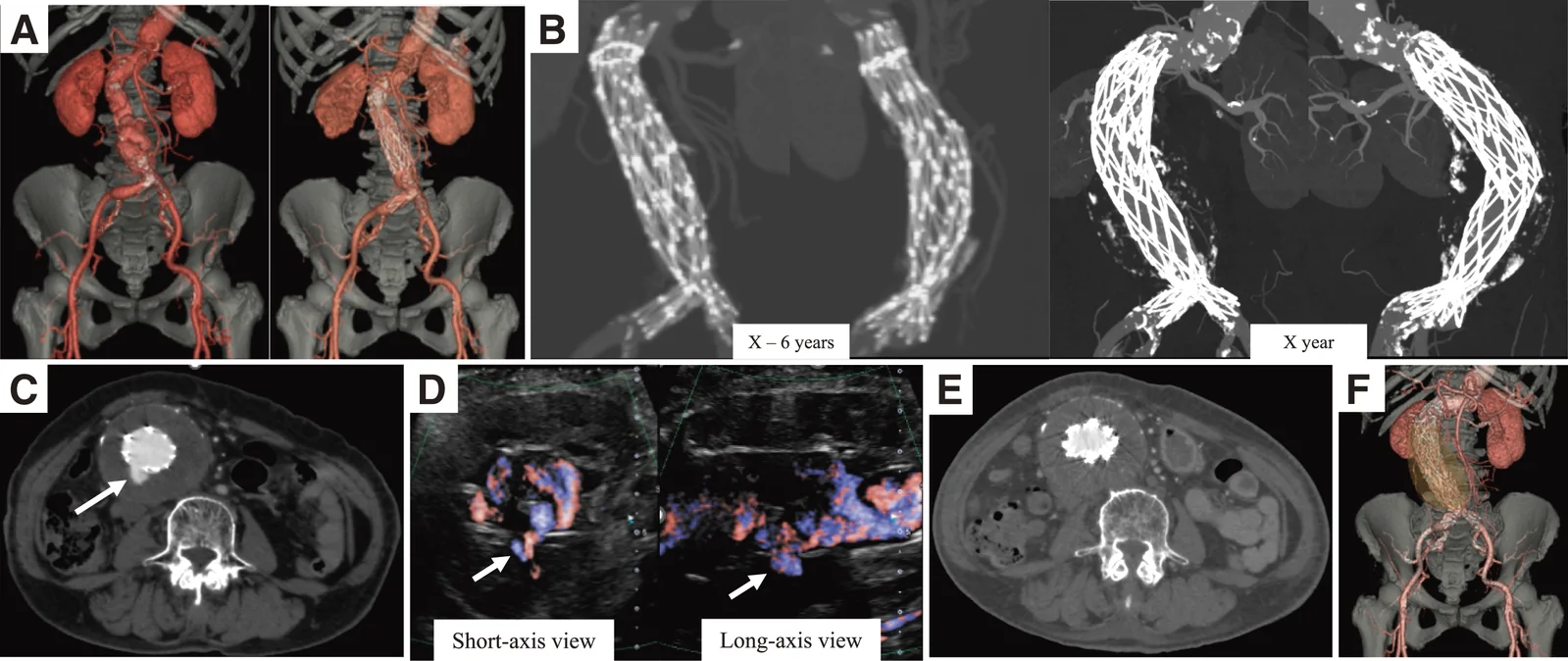
Clinical course and imaging findings of Case 1. (**A**) Preoperative 3-dimensional CTA and postoperative 3-dimensional CTA demonstrating successful EVAR with adequate overlap between the AFX2 (Endologix, Irvine, CA, USA) main body and proximal cuff. (**B**) Comparison of reconstructed CT images obtained after the initial EVAR and at the time of reintervention 6 years later, showing a change in stent-graft configuration, although definite component uncoupling was not identified. (**C**) Axial CTA obtained at the time of reintervention demonstrating contrast enhancement within the aneurysm sac adjacent to the component junction (arrow). (**D**) Duplex ultrasonography in short-axis and long-axis views demonstrating characteristic bidirectional (“to-and-fro”) flow near the component junction (arrows), providing an important diagnostic clue to type IIIa endoleak. (**E**) Postoperative axial CTA after focal relining demonstrating disappearance of the contrast enhancement previously observed adjacent to the component junction in panel **C**. (**F**) Postoperative 3-dimensional CTA after focal relining with additional VELA extension cuffs demonstrating complete exclusion of the aneurysm without residual endoleak. CT, computed tomography; CTA, computed tomography angiography; EVAR, endovascular aneurysm repair

After the initial postoperative CT assessment, the patient was followed at another institution because she lived far from our center. Serial CT images obtained during the intervening follow-up period were therefore not available at our institution. Six years after EVAR, aneurysm sac enlargement to 62 × 70 mm was detected, and the patient was referred back to our institution for further evaluation.

Laboratory investigations were unremarkable except for an elevated D-dimer level. Retrospective comparison of CT images obtained after the initial EVAR and at the time of reintervention demonstrated a change in stent-graft configuration, although definite component uncoupling was not identified (**Fig. 1B**). Axial CTA demonstrated contrast enhancement within the aneurysm sac adjacent to the junction between the proximal cuff and main body (**Fig. 1C**). Duplex ultrasonography demonstrated characteristic bidirectional (“to-and-fro”) flow within the aneurysm sac near the component junction (**Fig. 1D**), providing an important diagnostic clue to type IIIa endoleak.

Because the endoleak appeared localized to the modular junction and adequate proximal and distal landing zones remained, focal endovascular relining using additional extension cuffs was selected.

The procedure was performed under general anesthesia through bilateral percutaneous common femoral artery access using ultrasound guidance. Initial angiography confirmed a type IIIa endoleak originating from the junction between the main body and proximal cuff. Two VELA 34–100 mm extension cuffs were sequentially deployed to maximize overlap. Balloon molding was subsequently performed, and final angiography demonstrated complete resolution of the endoleak without residual leakage.

Postoperative axial CTA demonstrated disappearance of the contrast enhancement previously observed adjacent to the component junction (**Fig. 1E**), and 3-dimensional CTA confirmed complete exclusion of the aneurysm without residual endoleak (**Fig. 1F**). The postoperative course was uneventful, and the patient was discharged on postoperative day 11.

### Case 2

An octogenarian man had undergone EVAR 7 years earlier for a 51-mm infrarenal abdominal aortic aneurysm using an AFX2 main body (25–90/20–30 mm) with a VELA proximal extension cuff (28–95 mm). The overlap length measured 47 mm, and both completion angiography and postoperative CTA demonstrated satisfactory device positioning without endoleak (**Fig. 2A**).

**Fig. 2:**
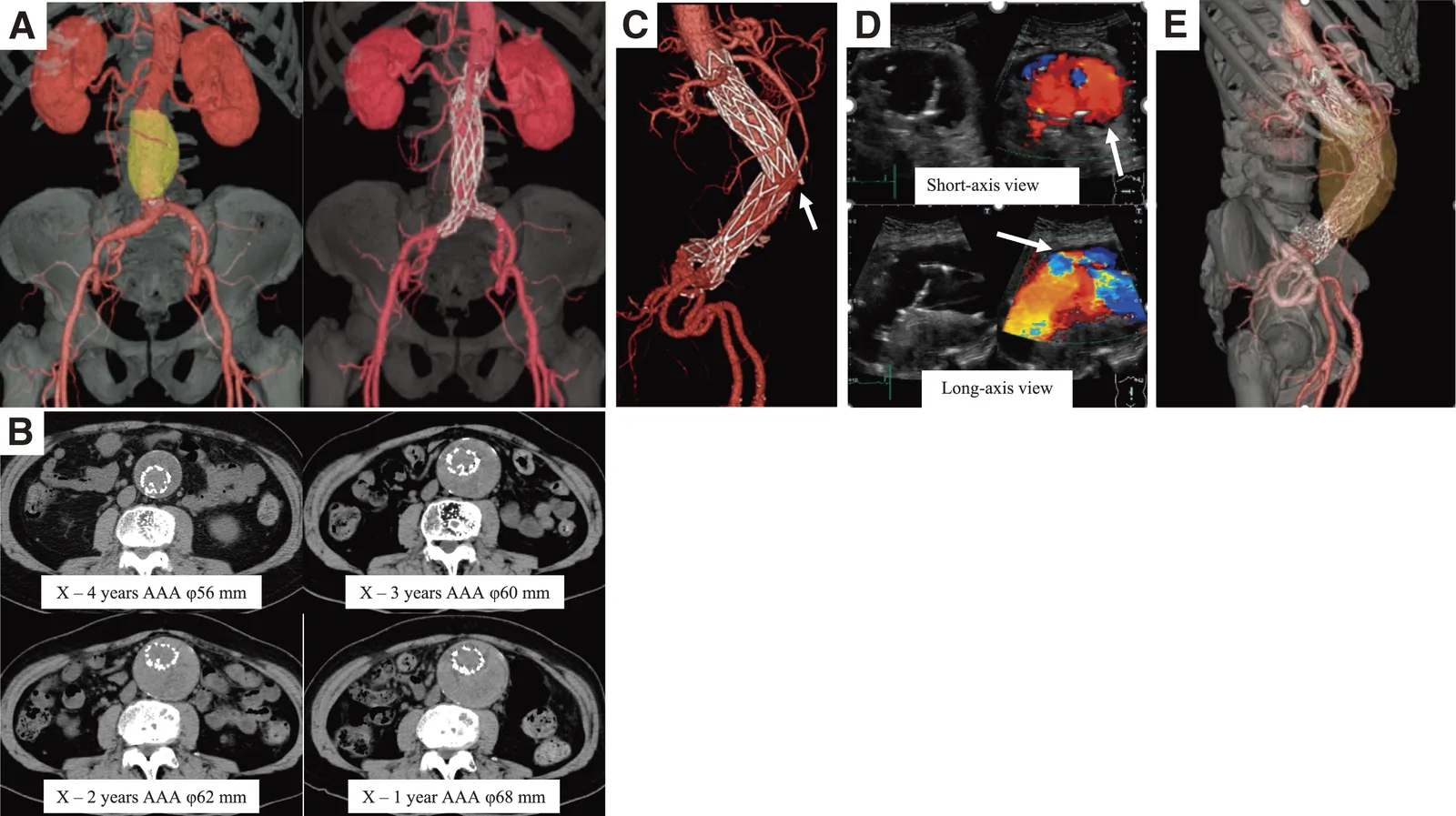
Clinical course of Case 2. (**A**) Preoperative 3-dimensional CTA and postoperative 3-dimensional CTA demonstrating successful EVAR with adequate overlap between the AFX2 (Endologix, Irvine, CA, USA) main body and proximal cuff. (**B**) Serial non-contrast CT images demonstrating gradual aneurysm sac enlargement during long-term follow-up. (**C**) Three-dimensional CTA demonstrating marked separation between the AFX2 main body and proximal cuff, consistent with component uncoupling. (**D**) Duplex ultrasonography in short-axis and long-axis views demonstrating characteristic bidirectional (“to-and-fro”) flow within the aneurysm sac. (**E**) Postoperative 3-dimensional CTA after complete endograft relining using the Endurant system demonstrating complete exclusion of the aneurysm without residual endoleak. CT, computed tomography; CTA, computed tomography angiography; EVAR, endovascular aneurysm repair

Serial surveillance non-contrast CT demonstrated gradual aneurysm sac enlargement from 56 mm at 3 years to 68 mm at 6 years after EVAR (**Fig. 2B**). Contrast-enhanced CTA performed 1 year before referral failed to demonstrate a definite endoleak, and observation was continued. Retrospective review of serial CT images demonstrated progressive lateral displacement of the AFX2 main body relative to the proximal cuff during follow-up, consistent with sideways displacement (**Fig. 3**).

**Fig. 3:**
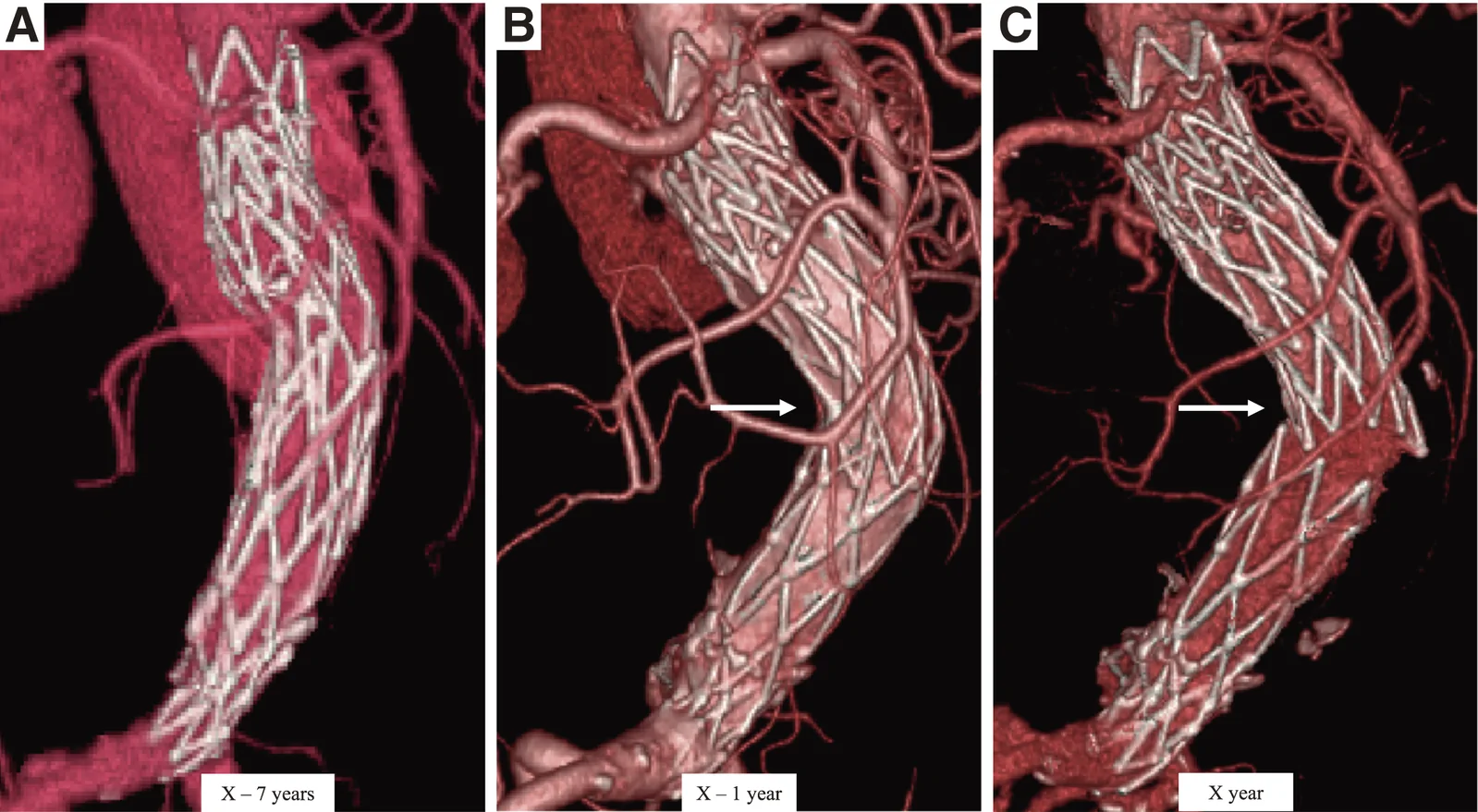
Longitudinal morphological changes of the AFX2 (Endologix, Irvine, CA, USA) components in Case 2. (**A**) Three-dimensional reconstructed CT obtained after the initial EVAR, 7 years before reintervention, demonstrating adequate overlap between the AFX2 main body and proximal cuff. (**B**) Three-dimensional reconstructed CT obtained 6 years after EVAR, 1 year before reintervention, demonstrating lateral displacement of the main body relative to the proximal cuff. (**C**) Three-dimensional CTA obtained 7 years after EVAR, at the time of reintervention, demonstrating marked component uncoupling after progressive sideways displacement. All images are shown at the same viewing angle (RAO 60°). White arrows indicate the direction of progressive lateral displacement of the main body relative to the proximal cuff. CT, computed tomography; CTA, computed tomography angiography; EVAR, endovascular aneurysm repair; RAO, right anterior oblique position

Despite the absence of an identifiable endoleak, the aneurysm further enlarged to 69 × 77 mm during the seventh postoperative year. Repeat CTA demonstrated subtle contrast enhancement within the aneurysm sac. Three-dimensional CTA clearly demonstrated marked separation between the main body and proximal cuff, consistent with intrasac component uncoupling (**Fig. 2C**). Duplex ultrasonography demonstrated characteristic bidirectional (“to-and-fro”) flow within the aneurysm sac (**Fig. 2D**), confirming the diagnosis of late type IIIa endoleak.

Because component separation was extensive, relining with an additional cuff alone was considered insufficient to achieve durable sealing. Therefore, complete endograft relining using the Endurant system (Medtronic, Santa Rosa, CA, USA) was planned.

Under general anesthesia, bilateral common femoral arteries were accessed percutaneously. Before device deployment, guidewire passage within the true endograft lumen was confirmed using an inflated balloon to avoid inadvertent passage through the endoskeleton. Initial angiography demonstrated a type IIIa endoleak. An Endurant main body (32–16–166 mm) was deployed, followed by placement of 16–20–199 mm and 16–20–124 mm iliac limbs into the left and right common iliac arteries, respectively. Completion angiography demonstrated complete exclusion of the aneurysm with no residual endoleak.

Postoperative CTA confirmed successful relining with complete disappearance of the endoleak and no evidence of persistent aneurysm sac perfusion (**Fig. 2E**). The patient was discharged uneventfully.

## Discussion

Late type IIIa endoleak caused by separation at the modular junction is a recognized device-related complication of the AFX/AFX2 platform.^3–6)^ The present cases provide additional clinical observations of this recognized failure mode rather than evidence of a fundamentally new mechanism. Their clinical relevance lies in the long interval from implantation to diagnosis, the different imaging presentations between the 2 patients, and the longitudinal demonstration of progressive device deformation in Case 2 despite adequate initial overlap.

Type IIIa endoleak results from separation between modular endograft components and exposes the aneurysm sac to systemic arterial pressure, substantially increasing the risk of aneurysm enlargement and rupture.^1,2,5)^ Therefore, current guidelines recommend prompt treatment once a type III endoleak is diagnosed.^1,2)^ The AFX2 endograft has an endoskeleton design and is often used with a proximal extension cuff, creating a modular junction at which late component separation can occur.^3–7)^

Hoshina et al. reported acceptable midterm outcomes of AFX2 endografts used with aortic cuffs but identified sideways displacement in 4 migration cases, including 1 associated with type IIIa endoleak, and emphasized the importance of surveillance for endograft deformation.^3)^ Ueda et al. subsequently reported complete uncoupling 4 years after AFX2 implantation despite adequate initial overlap; retrospective review of serial CT images suggested progressive displacement of the main body and cuff, and the authors highlighted the usefulness of 3-dimensional CT assessment of device displacement.^4)^ Skibba et al. also reported late component uncoupling associated with progressive changes in endograft configuration, including sideways displacement and loss of component overlap.^5)^ Ko et al. reported late type III endoleak after loss of component overlap in an AFX2 endograft and emphasized careful surveillance for late morphological changes, particularly in patients with large aneurysms and tortuous anatomy.^6)^

Importantly, an overlap of 47 mm was achieved in both patients. Before the August 2018 revision of the AFX2 instructions for use, the recommended proximal component overlap was 30–40 mm. In August 2018, the manufacturer issued an Important Safety Update revising the sizing guidance to require an overlap exceeding the aneurysm radius plus 20 mm.^8)^ The index procedure in Case 2 was performed shortly before this revision, whereas that in Case 1 was performed shortly thereafter. Based on the initial aneurysm diameters of 47 and 51 mm, the corresponding overlap lengths required according to the revised criterion were 43.5 and 45.5 mm in Cases 1 and 2, respectively. Thus, the 47-mm overlap achieved in both patients exceeded the earlier 30–40-mm recommendation and also satisfied the revised patient-specific criterion. Nevertheless, late junctional failure occurred in both cases. These findings, together with previous reports, indicate that adequate initial overlap alone does not guarantee durable junctional stability.^3–6,9)^

The mechanism of late junctional failure is likely multifactorial and cannot be explained by overlap length alone. Previous reports have described migration, sideways displacement, and progressive conformational changes of AFX/AFX2 components.^3–6)^ Ueda et al. discussed anteroposterior angulation of the AFX main body of more than 30° as a possible factor associated with sideways displacement.^4)^ In the present cases, the angle between the main body and proximal cuff was approximately 35° in Case 1 and 39° in Case 2. Although these angles were within the manufacturer’s recommended limit of ≤60°, the observations reported by Ueda et al. suggest that angulation may still have contributed to progressive component deformation. However, angulation alone is unlikely to explain the late junctional failure in these cases. Importantly, the proximal end of the main body remained within the aneurysm sac in both patients. In Case 2, retrospective review of serial CT images demonstrated progressive sideways displacement of the main body relative to the proximal cuff before complete uncoupling became apparent (**Fig. 3**). These findings suggest that the intrasac position of the main body and progressive changes in component configuration may have contributed to loss of effective overlap over time. In Case 1, a change in stent-graft configuration was observed when the initial and pre-reintervention CT images were compared, but definite component uncoupling was not demonstrated on CT. Thus, the pathway to late junctional failure may differ among patients and likely reflects an interaction between device configuration, patient-specific anatomy, and progressive morphological change.

The 2 cases also illustrate different imaging manifestations of late junctional failure and the complementary roles of imaging modalities. In Case 1, definite component uncoupling was not demonstrated on CTA. Nevertheless, duplex ultrasonography clearly demonstrated characteristic bidirectional (“to-and-fro”) flow near the component junction, providing an important diagnostic clue to type IIIa endoleak, which was subsequently confirmed by intraoperative angiography. Duplex ultrasonography can provide complementary information for endoleak detection during EVAR surveillance.^10)^ Case 1 therefore illustrates the potential diagnostic value of duplex ultrasonography when morphological evidence of component separation is inconclusive on CT. In contrast, in Case 2, serial CT demonstrated progressive sac enlargement, and retrospective comparison revealed progressive sideways displacement before 3-dimensional CTA eventually demonstrated complete uncoupling. Longitudinal comparison of CT images may therefore be important for identifying progressive changes in endograft configuration.^3–6)^

Plain radiography may also provide useful information regarding changes in component position and separation during long-term surveillance. However, plain radiography was not performed in either patient. Furthermore, in Case 1, after the initial postoperative CT assessment, subsequent follow-up was performed at another institution because the patient lived far from our center, and serial CT images obtained during the intervening period were not available to us. This limited longitudinal assessment of the stent-graft configuration in Case 1. These cases suggest that surveillance after AFX2 implantation should assess not only aneurysm sac diameter and endoleak but also interval changes in component position and configuration when serial imaging is available.

The treatment methods used in these cases are established endovascular options rather than novel techniques.^5,9)^ The choice of strategy was based on the extent of junctional failure and the available sealing zones. In Case 1, the leak was localized to the modular junction and adequate proximal and distal landing zones remained; therefore, focal relining with additional VELA extension cuffs was considered sufficient. In Case 2, separation was extensive, and bridging with an additional cuff alone was considered less likely to provide durable exclusion; complete relining with an Endurant endograft was therefore selected. This distinction illustrates the practical importance of tailoring secondary intervention to the morphology and extent of component failure.

Long-term durability after AFX2 EVAR requires careful surveillance and timely secondary intervention.^3,4,6,9)^ The present cases further emphasize that surveillance after AFX2 implantation should continue lifelong because clinically important junctional failure may occur many years after apparently successful implantation.^3,4,6,9)^ In patients with progressive sac enlargement, comparison with prior imaging should include careful assessment for subtle changes in device configuration even when a definite endoleak is not initially visible.^3–6)^

This report has several limitations. It describes only 2 patients from a single institution and cannot establish the incidence or causal mechanism of late component uncoupling. The relationship between the observed anatomical and configurational features and subsequent component failure therefore remains inferential. Plain radiography was not performed in either patient. In addition, serial CT images obtained during the intervening follow-up period were not available for Case 1, limiting longitudinal assessment of component position and configuration. Nevertheless, Case 2 provides serial imaging evidence consistent with previously reported progressive sideways displacement,^3–6)^ whereas the different diagnostic pattern in Case 1 underscores the complementary value of duplex ultrasonography when CTA does not clearly demonstrate component uncoupling.^10)^

## Conclusion

Late type IIIa endoleak may occur years after AFX2 implantation despite adequate initial component overlap. Progressive endograft deformation, including sideways displacement, may contribute to late junctional failure in some patients. Longitudinal assessment of device configuration, together with complementary use of duplex ultrasonography when CTA is inconclusive, may facilitate recognition of late junctional failure. Lifelong surveillance and treatment tailored to the extent of component failure remain essential after AFX2 EVAR.
